# Retraction Note: Microarray analysis for differentially expressed genes of patients undergoing total knee arthroplasty with ischemia preconditioning

**DOI:** 10.1186/s13018-015-0182-z

**Published:** 2015-03-26

**Authors:** Jianguang Wang, Zhengdong Cai, Junjian Liu

**Affiliations:** The Affiliated Shanghai No.10 People’s Hospital, Nanjing Medical University, Shanghai, 200072 China; Department of Orthopedics, Shanghai Tenth People’s Hospital, Tongji University School of Medicine, Shanghai, 200072 China; The Affiliated Shanghai No.1 People’s Hospital, Nanjing Medical University, Shanghai, 200080 China; Department of Orthopedics, Shanghai First People’s Hospital, Shanghai Jiao Tong University, Shanghai, 200080 China; Department of Orthopedics, Shanghai First People’s Hospital, No.100 Haining Road, Hongkou District, Shanghai, 200080 China

## Retraction

The Publisher and Editor regretfully retract this article [1] because the peer-review process was inappropriately influenced and compromised. As a result, the scientific integrity of the article cannot be guaranteed. A systematic and detailed investigation suggests that a third party was involved in supplying fabricated details of potential peer reviewers for a large number of manuscripts submitted to different journals. In accordance with recommendations from COPE we have retracted all affected published articles, including this one. It was not possible to determine beyond doubt that the authors of this particular article were aware of any third party attempts to manipulate peer review of their manuscript.
